# Secondary Metabolites from Marine Sponges of the Genus *Oceanapia*: Chemistry and Biological Activities

**DOI:** 10.3390/md20020144

**Published:** 2022-02-16

**Authors:** Meng-Juan Xu, Lin-Jing Zhong, Jun-Kun Chen, Qing Bu, Lin-Fu Liang

**Affiliations:** College of Materials Science and Engineering, Central South University of Forestry and Technology, Changsha 410004, China; shine0805@163.com (M.-J.X.); zljswan120101@163.com (L.-J.Z.); cjk_kg@outlook.com (J.-K.C.); 17355249596@163.com (Q.B.)

**Keywords:** *Oceanapia*, sponge, secondary metabolites, biological activities

## Abstract

In this review, we summarized the distribution of the chemically investigated *Oceanapia* sponges, including the isolation and biological activities of their secondary metabolites, covering the literature from the first report in 1989 to July 2019. There have been 110 compounds reported during this period, including 59 alkaloids, 33 lipids, 14 sterols and 4 miscellaneous compounds. Besides their unique structures, they exhibited promising bioactivities ranging from insecticidal to antibacterial. Their complex structural characteristics and diverse biological properties have attracted a great deal of attention from chemists and pharmaceuticals seeking to perform their applications in the treatment of disease.

## 1. Introduction

Marine sponges, living in harsh marine conditions from tropic to polar regions, offer an enormous source of natural products bearing unique structures and significant bioactivities, making them ideal candidates for drug discovery projects [1]. Of them, the animals belonging to the genus *Oceanapia* (phylum, Porifera; class, Demospongiae; subclass, Heteroscleromorpha; order, Haplosclerida; family, Phloeodictyidae) have proven to be a biochemical warehouse for secondary metabolites, such as alkaloids, lipids, sterols, etc. It is particularly interesting that these compounds exhibited a wide range of biological features ranging from antibacterial and cytotoxic to ichthyotoxic activities [2]. In order to better understand the natural products from this genus, there would be a demand for a review.

**Figure 1:**
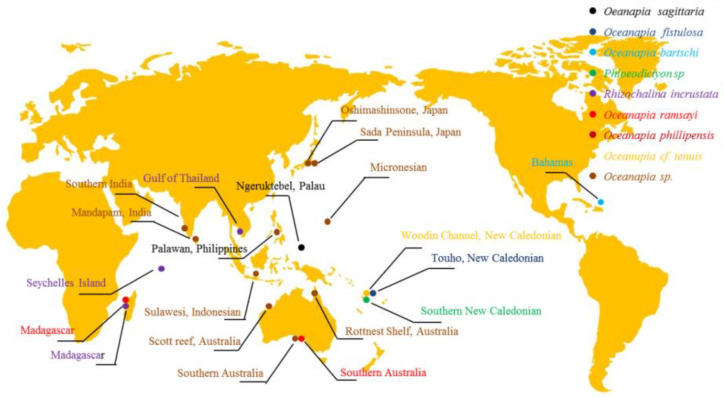
Global distribution of the chemically investigated marine *Oceanapia* sponges according to their species.

Notably, several other generic names (*Phloeodictyon* Carter, 1882; *Rhizochalina* Schmidt, 1870; *Biminia* Wiedenmayer, 1977; *Foliolina* Schmidt, 1870) are now considered as synonyms for *Oceanapia* [3]. However, there is no associated reference on secondary metabolites of two genera *Biminia* and *Foliolina* listed in *SciFinder Scholar*. Therefore, this review covers topics on three nominal genera, *Oceanapia*, *Phloeodictyon* and *Rhizochalina,* covering different types of compounds, with a literature survey from 1989 to July 2019. During this period, 110 compounds have been reported, including 59 alkaloids, 33 lipids, 14 sterols and 4 other miscellaneous compounds. More than eight species of *Oceanapia* sponges have been chemically investigated including *Oeanapia sagittaria*, *Oceanapia fistulosa*, *Oceanapia bartschi*, *Phloeodictyon* sp., *Rhizochalina incrustata*, *Oceanapia ramsayi*, *Oceanapia phillipensis*, *Oceanupia* cf. *tenuis* and *Oceanapia* sp. The global distribution of the chemically investigated marine *Oceanapia* sponges according to their species is shown in Figure 1.

## 2. Alkaloids

Alkaloids were encountered most frequently. They can be classified as pyridoacridine alkaloids, quinolizidine alkaloids, sesquiterpene alkaloid, phloeodictine alkaloidalkaloids, bromotyrosine alkaloids, indole alkaloids and nucleotide alkaloids, according to their skeletons.

### 2.1. Pyridoacridine Alkaloids

**Figure 2:**
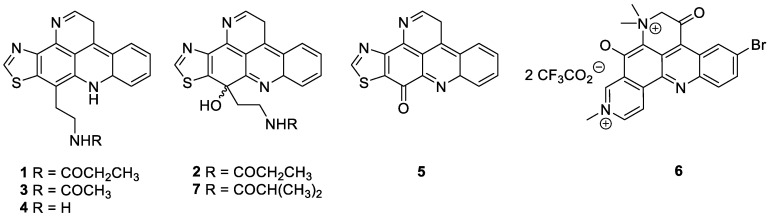
The chemical structures of pyridoacridine alkaloids **1**–**7**.

Faulkner’s group found the sponge *Oceanapia sagittaria* from Palau contained two pyridoacridine alkaloids dercitamide (**1**) and sagitol (**2**) (Figure 2). Of them, **2** was the first pyridoacridine alkaloid from a marine sponge in which the aromatic system had been disrupted. Interestingly, **2** could be obtained by autoxidation of **1**. Faulkner et al. suggested that **2** was not an artifact that was supported by CD measurements. [4]. Two years later, Proksch et al. reported three pyridoacridine alkaloids kuanoniamine C (**1**), kuanoniamine D (**3**) and *N*-deacetylkuanoniamine C (**4**) were afforded by the Micronesian sponge *Oceanapia* sp. [5]. It may be worthy to point out that the structures of dercitamide and kuanoniamide C were established to be identical by Faulkner and his co-workers [6]. Herein, the same numbering was assigned for these two different nomenclative compounds. Proksch et al. performed many bioassays for these three alkaloids. When incorporated into an artificial diet, compounds **1** and **3** exhibited insecticidal activity toward neonate larvae of the polyphagous pest insect *Spodoptera littoralis* (LC_50_ of 156 and 59 ppm, respectively). Both compounds also showed toxicity in the brine shrimp lethality test with LC_50_ values of 37 and 19 µg/mL, respectively. Although the *N*-deacyl derivative **4** did not show any remarkable effect in either of the abovementioned bioassays, it appeared to be active in the cytotoxic biotests against two human cell lines. The IC_50_ of **4** was 1.2 µg/mL toward HeLa cells and 2.0 μg/mL toward MONO-MAC 6 cells. In receptor binding assays, compound 3 showed affinity to A_1_- and A_2A_-adenosine receptors with *K*_i_ values of 2.94 and 13.7 µM, respectively. Compound **1** was less active than its homologue **3**, whereas the *N*-deacyl derivative **4** showed no affinity toward adenosine receptors. In addition, compounds **1**, **3** and **4** exhibited moderate affinity to benzodiazepine binding sites of GABA_A_ receptors [5]. Meanwhile, Proksch et al. explored the distribution of compounds **1** and **3** in the sponge *Oceanapia* sp., as well as its ecological implications. It was found that the secondary metabolites **1** and **3** showed a sharp increase from the basal root to the capitum. The feeding assays against the spongivorous angelfish *Pomacanthus imperator* showed that **1** and **3** significantly deterred feeding by natural assemblages of reef fishes at fistule concentrations, confirming their role as defensive agents [7].

Kijjoa and his co-workers reported another specimen, *O. sagittaria* from the Gulf of Thailand, afforded kuanoniamine C (**1**) and its relative compound kuanoniamine A (**5**). In this study, compounds **1** and **5** were evaluated for cytotoxic effects against five human tumor cell lines MCF-7 (ER+), MDA-MB-231 (ER-), SF-268, NCI-H460 and UACC-62, and one human non-tumor cell line, MRC-5, by the SRB method. Compound **5** was found to be a potent growth inhibitor of all tumor and the non-tumor cell lines while **1** was less potent but showed high selectivity toward the estrogen-dependent (ER+) breast cancer cell line MCF-7. Furthermore, **5** was shown to be a more potent inhibitor of DNA synthesis than **1**. It was also found that **5** caused an extensive reduction in the MCF-7 cells in the G2/M phase as well as an increase in the apoptotic cells [8].

Bioassay-guided fractionation of the MeOH extract of an Australian sponge *Oceanapia* sp. performed by Carroll’s group, using the aspartyl semialdehyde dehydrogenase (ASD) to detect antibacterial activity, led to the discovery of a bright blue compound, petrosamine B (**6**). It was found **6** was a weak inhibitor of ASD with an IC_50_ of 306 µM [9]. In Ibrahim’s investigation of the Indonesian sponge *Oceanapia* sp., sagitol C (**7**) together with the two abovementioned compounds **1** and **2** were disclosed. The cytotoxic effect of **7** was tested against mouse lymphoma (L5178Y), rat brain (PC12) and human cervix (Hela) cell lines. It exhibited 93%, 88% and 76% growth suppression against the tested cell lines at a concentration of 24.6 μM and 81%, 74% and 37% at a concentration of 12.3 μM with ED_50_ values of 0.7, 0.9 and 2.3 µM, respectively [10]. 

### 2.2. Quinolizidine Alkaloids 

**Figure 3:**
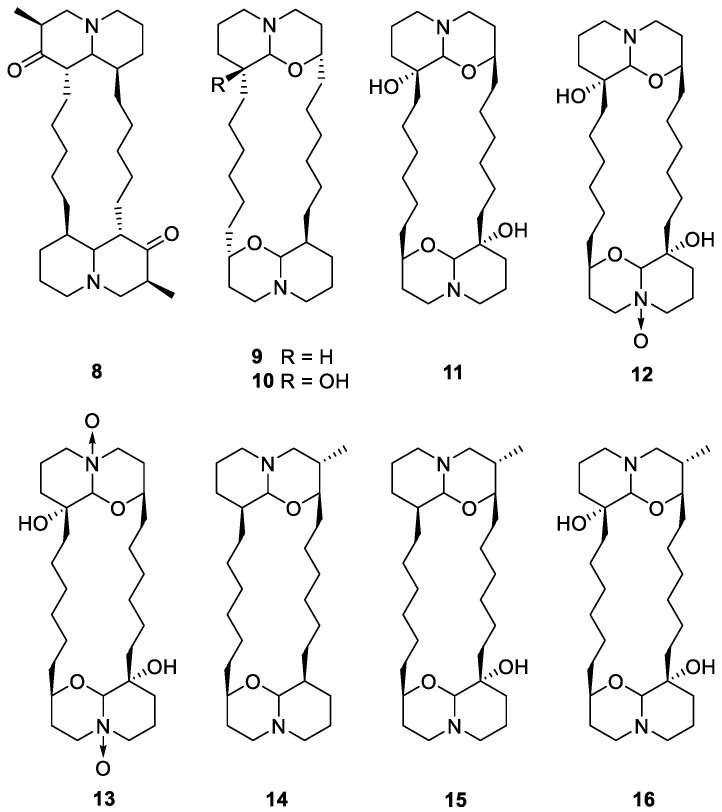
The chemical structures of quinolizidine alkaloids **8**–**16**.

A bisquinolizidine alkaloid, petrosin (**8**), and a series of *bis*-1-oxaquinolizidine alkaloids, xestospongins C–J (**9**–**16**) (Figure 3), were all obtained by Singh and his partners from the ethyl acetate extract of the sponge *Oceanapia* sp., which was collected from the southern coast of India. The relative stereochemistry of **8** was established by single-crystal X-ray analysis as 1S*,2R*,4R*,9S*,15R*,17R*,22S*,23S*. Compounds **9** and **10** were found to be active against several pathogens such as *Cryptococcus neoformans*, *Aspergillus funigatus*, *Candida albicans* and *Aspergillus niger* [11]. 

### 2.3. Sesquiterpene Alkaloid

**Figure 4:**
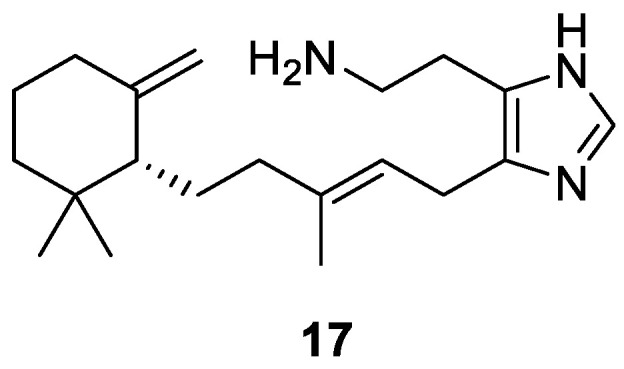
The chemical structure of sesquiterpene alkaloid **17**.

Faulkner’s group disclosed the major metabolite of the *Philippine* sponge *Oceanapia* sp. was the antimicrobial alkaloid oceanapamine (**17**) (Figure 4), isolated as trifluoroacetate (TFA) salt. The structure of **17** consisted of a monocyclic sesquiterpene attached to a histamine residue, representing the sole sesquiterpene–alkaloid hybrid from the genus *Oceanapia*. Compared with related model compounds, the absolute configuration of **17** was assigned as 6*R*. The TFA salt of **17** was screened rather broadly but only exhibited antimicrobial activity. In the standard disk (6-mm) assay, **17** inhibited *Bacillus subtilis* and *Escherichia coli* at 25 µg/disk, *Staphyloccocus aureus* and *C. albicans* at 50 μg/disk and *Pseudomonas aerivginosa* at 100 μg/disk [12]. 

### 2.4. Phloeodictine Alkaloids

**Figure 5:**
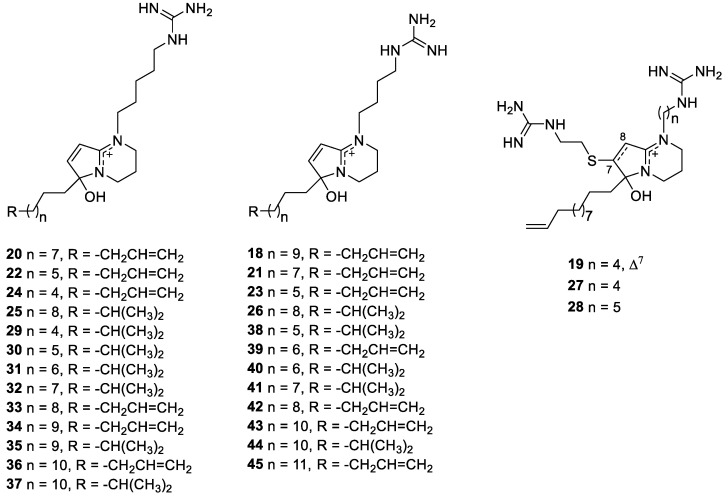
The chemical structures of phloeodictine alkaloids **18**–**45**.

The phloeodictyne framework was characterized by a fused alkaloidal skeleton, 1,2,3,4-tetrahydropyrrolo[1,2-*a*]pyrimidinium, bearing a variable-length alkyl (or alkenyl) side chain at C-6 and a four/five methylene chain ending in a guanidine group at *N*-1, while a thioethylguanidine chain may have been present at C-7 or not. Kourany-Lefoll et al. first reported this group of alkaloids in the haplosclerid sponge *Phloeodictyon* sp. living in deep New Caledonian waters. Included were the pure compounds phloeodictines A (**18**) and B (**19**) and the inseparable mixtures of phloeodictines A1 (**20**) and A2 (**21**), A3 (**22**), A4 (**23**) and A5 (**24**), A6 (**25**) and A7 (**26**) and C1 (**27**) and C2 (**28**) (Figure 5). Compounds **18** and **19** had been tested against several bacteria using the standard microdilution plate assay and revealed to have potent activity with the following respective MICs (μg/mL): *S. aureus* (1, 3), *E. coli* (1, 30), *P. aeruginosa* (10, >30) and *Streptococcus fecalis* (5, >15). On the other hand, the mixtures A (**20**+**21**), B (**22**+**23**+**24**), C (**25**+**26**) and D (**27**+**28**) were found to possess a wider spectrum of antibacterial activity (respective MICs, μg/mL): *S. aureu* (3, 30, 1, 3), *E. coli* (3, 30, 3, >30), *P. aeruginosa* (30, >30, 30, >30), *Clostridium perfringens* (30, >30, 1, >100), *Bucteroides frugilis* (10, ~30, 3, >100) and *Peptococcus assacckarolyticus* (10, >30, 3, >100). Furthermore, compounds **18**, **19** and mixtures A–D also exhibited in vitro cytotoxicity toward KB human nasopharyngeal carcinoma cells with IC_50_ values of 1.5, 11.2, 2.2, 3.5, 0.6 and 1.8 μg/mL, respectively [13,14]. Ten years later, Snider and his co-worker completed the first synthesis of (±)-**21** [15].

Phloeodictines were also found to be components of the sponge *Oceanapia* [= *Phloeodictyon*] *fistulosa* in New Caledonian shallow waters by Mancini et al. They were clarified as a wide structural variety including the known **18**–**28** and new analogues **29**–**45** as corresponding mixtures. The complexity of the mixtures, and the very similar behavior of their components, prevented their isolation in pure form. However, crude mixtures and HPLC-enriched fractions were suitable for bioassays and proved to be active against chloroquine-resistant *Plasmodium falciparum*, with IC_50_ values ranging from 0.6 to 6 µM, while cytotoxicity against the human A-549 cell line was low. These biological data might serve to illustrate preliminary structure–activity relationships: 1. The length of the C-6 chain had a greater influence on the bioactivity level than the nature of its terminal portion; 2. methylation of the guanidine moiety lowered the activity. This study showed good prospects for these alkaloids as leads for novel antimalarial agents [16].

### 2.5. Bromotyrosine Alkaloids

**Figure 6:**
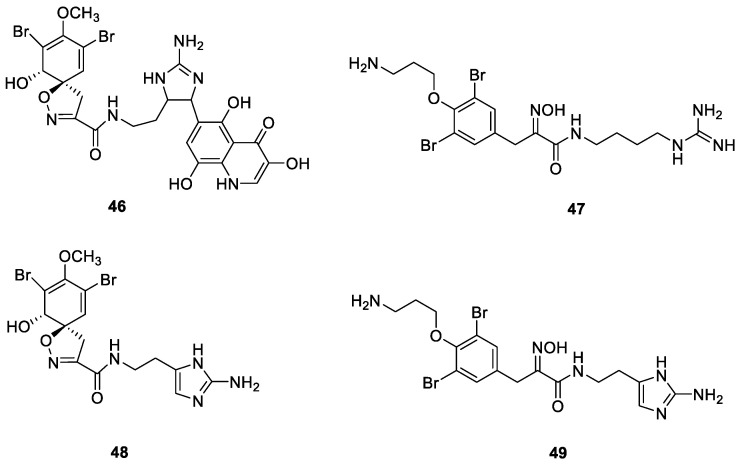
The chemical structures of bromotyrosine alkaloids **46**–**49**.

Four bromotyrosine alkaloids **46**–**49** (Figure 6) were all isolated from an Australian non-verongid sponge *Oceanapia* sp. by Bewley’s group. Among them, **46** contained an unprecedented imidazolyl-quinolinone substructure attached to a bromotyrosine-derived spiro-isoxazoline. In the bioassay, compounds **46**–**49** inhibited mycothiol *S*-conjugate amidase by 50% at 2, 100, 3 and 37 mM, respectively. These four alkaloids represented the first examples of natural products that inhibited an enzyme central to a mycothiol-dependent detoxification pathway found in mycobacteria [17].

### 2.6. Indole Alkaloids

**Figure 7:**
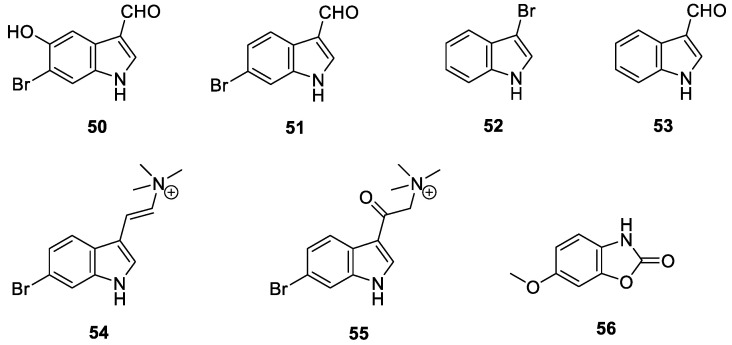
The chemical structures of indole alkaloids **50**–**56**.

6-Bromo-5-hydroxy-3-indolecarboxyaldehyde (**50**) along with two other brominated indoles 6-bromo-3-indolecarboxyaldehyde (**51**) and 3-bromoindole (**52**) (Figure 7) were discovered in the Caribbean sponge *Oceanapia bartschi* by Fattorusso’s group [18]. Their structurally related non-brominated indole 3-formylindole (**53**) was disclosed in the Thai sponge *O. sagittaria* [8]. Crews’ group investigated the Indonesian sponge *Oceanapia* sp., leading to another two brominated indoles, 6-Br-conicamin (**54**) and 6-Br-8-keto-conicamin A (**55**). Meanwhile, they synthesized **55** in this study. In the bioassay, the low micromolar in vitro activity of **55** against the PANC-1 cell line (IC_50_ 1.5 mM for the natural product vs. 4.1 mM for the synthetic material) was exciting, which was similar to that of the clinical therapeutics (5FU: IC_50_ = 7.0 mM; gemcitabine: IC_50_ = 0.02 mM). Furthermore, ten additional analogs were prepared for further study on the structure–activity relationship. The continued study indicated that the quaternary amine functionality and bromination of the indole ring of **55** were critical for activity against PANC-1 [19]. The Indian sponge *Oceanapia* sp. afforded coixol (**56**), an active compound in the brine shrimp assay (LC_50_ = 52.93 ± 6.48 ppm). This was the first report of coixol from a marine source [20].

### 2.7. Nucleotide Alkaloids

**Figure 8:**
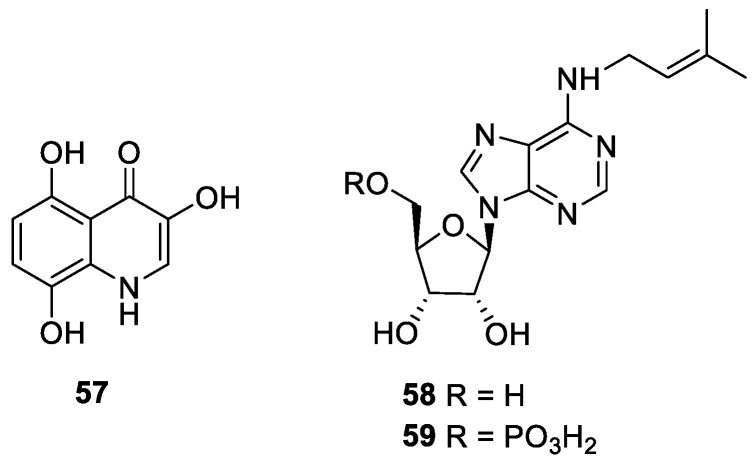
The chemical structures of nucleotide alkaloids **57**–**59**.

In the study of an Australian sponge *Oceanapia* sp., uranidine (**57**) (Figure 8) was discovered as one component of the major alkaloids [17]. Very recently, *N*^6^-isopentenyladenosine (i^6^A, **58**), along with *N*^6^-isopentenyladenosine 5ʹ-monophosphate (i^6^AP, **59**), was isolated from a Japanese sponge *Oceanapia* sp. This was the first report of i^6^A (**58**) and i^6^AP (**59**) from a marine sponge. In the cytotoxic biotest, **58** exhibited cytotoxic activity against HeLa cells with an IC_50_ value of 2.1 µM. On the other hand, **59** was inactive at a concentration of 50 μM. Further observations demonstrated that the cell cycle was arrested at the G1 phase by **58**, which indicated targeting of the Akt/NF-κB pathway [21].

## 3. Lipids

Lipids were the second-largest group of the *Oceanapia* secondary metabolites. They could be divided into sphingolipids, ceramides and cerebrosides, dithiocyanates and polyacetylenes, according to their structure features.

### 3.1. Sphingolipids

**Figure 9:**
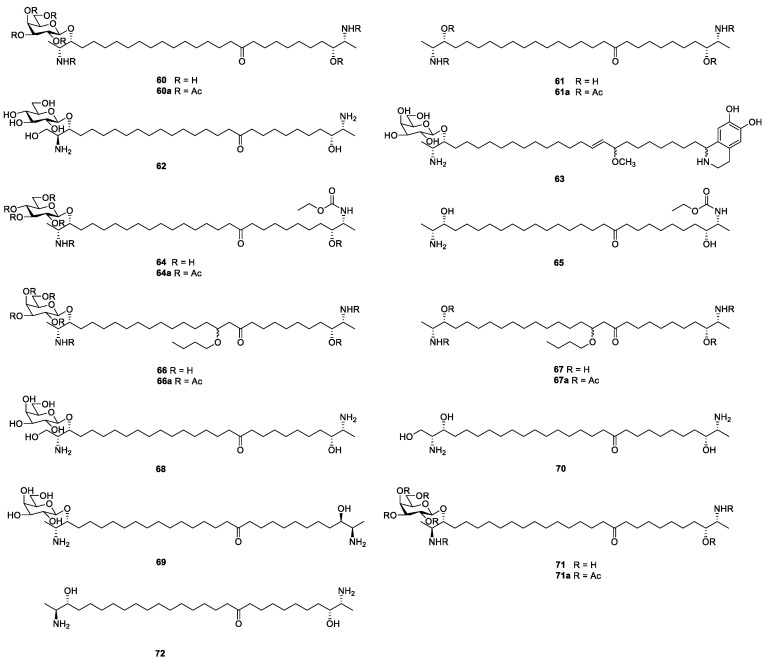
The chemical structures of sphingolipids **60**–**72**.

An antimicrobial galactopyranosyl pseudodimeric *α*,*ω*-bipolar sphingolipid, rhizochalin (**60**) (Figure 9), was isolated by Makarieva et al. from the sponge *Rhizochalina incrustata* near the north-west shore of Madagascar Island [22]. This was the first report of sphingolipids in the sponge of the genus *Oceanapia*. The effects of **60** on cell membranes were studied in *Ehrlich ascites* cells, spleen lymphocytes and erythrocytes, and phospholipid liposomes, respectively. At 10–100 mg/mL, this compound altered membrane permeability. These effects might be related to the cytostatic activity of **60** [23]. Ten years later, Molinski and his co-workers determined the absolute stereochemistry of (-)-rhizochalin (**60**) as 2*R*,3*R*,26*R*,27*R* by application of a general CD method based on the superposition of additive exaction couplings in tetrabenzoyl derivatives of *bis*-amino alkanols [24]. In Gaydou et al.’s study of a specimen of *Oceanapia ramsayi* collected at Itampolo on the west coast of Madagascar, rhizochalin (**60**) was found together with its corresponding aglycone rhizochalinin (**61**), which were both identified by their corresponding peracetates **60a** and **61a** [25]. A series of cytotoxic bioassays for **60** and **61** against different cell lines including the mechanisms had been carried out. Fedorov et al. reported **60** and **61** were cytotoxic against JB6 P+ Cl41, HeLa and THP-1 cell lines with IC_50_ values ranging from 2.8 to 22.1 µM. A more in-depth study revealed **60** inhibited the EGF-induced transformation of JB6 P+ Cl41 cells in a dose-dependent manner [26]. Stonik and Kwak observed **60** and **61** induced apoptosis of HL-60 cells, of which the latter showed a stronger ability. Further detailed study showed the usual mitochondrial membrane permeability changes and the decrease in protein levels of procaspases-8, -9 and -3 correlated with their apoptotic activity [27]. Choi et al. disclosed **61** induced apoptosis via activation of AMP-activated protein kinase in HT-29 colon cancer cells [28].

Oceanapiside (**62**), a new *bis*-*α*,*ω*-amino alcohol glycoside from the marine sponge *Oceanapia phillipensis* collected in southern Australia, was reported by Molinski’s group [29]. Soon after, the absolute stereochemistry of **62** was assigned 2*S*,3*R*,26*R*,27*R* by analysis of CD spectra of its perbenzoate [30]. Compound **62** exhibited significant antifungal activity against the fluconazole-resistant yeast *Candida glabrata* with an MIC of 10 µg/mL in broth dilution experiments [29]. In addition, in vitro antifungal activity of a series of *α*,*ω*-bifunctionalized amino alcohols derived from **62** against *C. glabrata* was measured. The dimeric bifunctionalized lipids exhibited activity about 10-fold higher than D-sphingosine, which was a larger factor than expected from the simple additive effects of vicinal amino alcohol groups [31]. It may be worth pointing out that the application of a combined method including micromolarscale Baeyer–Villiger oxidation and LC–MS interpretation by Makarieva et al. led to a revision of the structure of **62**, in which the placement of the keto group should be at C-18 rather than C-11 [32]. Oceanalin A (**63**), a unique hybrid *α*,*ω*-bifunctionalized sphingoid tetrahydroisoquinoline *β*-glycoside, was discovered in the sponge *Oceanapia* sp. collected off the northwest coast of Australia. Its absolute structure 2*R*,3*R* was elucidated by chemical correlation with the known rhizochalin **60**. Compound **63** exhibited in vitro antifungal activity against *C. glabrata* with an MIC of 30 µg/mL [33].

Rhizochalin A (**64**), the fourth representative of two-headed glycosphingolipids, was isolated as its peracetate (**64a**) from the sponge *R. incrustata* collected in the Seychelles Islands. Based on the chemical correlation, the absolute configuration 2*R*,3*R*,26*R*,27*R* of **64** was determined to be the same as that of **60**. It might be worth pointing out that **64a** was the first example of a natural product among known sphingolipids, including the family of two-headed sphingolipids, that contained the rare *N*-alkyl carbamoyl group [34]. Soon after, rhizochalinin A (**65**), the aglycone of rhizochalin A (**64**), was discovered in the former specimen. Compound **65** exhibited antileukemic activity against human leukemia THP-1 cells (IC_50_ = 7.5 µM) [35]. Two new, two-headed sphingolipid-like compounds, rhizochalin B (**66**) and its aglycone rhizochalinin B (**67**), were obtained as their corresponding peracetates (**66a**, **67a**) from the marine sponge *Oceanapia* sp. collected near the Scott reef (northwest of Australia). They differed from classical sphingolipids in the *α*,*ω*-position of the basic groups, resembling the polar ends of sphingoid bases, and often contained a terminal methyl group instead of the hydroxymethyl group. Notably, the stereochemistry of C-16 in compounds **66** and **67** remained unknown [36].

From the sponge *R. incrustata* collected in Madagascar, two new representatives of two-headed glycosphingolipids, rhizochalins C (**68**) and D (**69**), were discovered. Based on the analysis of ^1^H NMR data and CD spectra of their corresponding perbenzoates, the absolute configurations of **68** and **69** were assigned 2*R*,3*R*,26*R*,27*R* and 2*R*,3*R*,27*R*,28*R*, respectively. In contrast to the regular 2*S*,3*R*-configuration found in normal sphingoid bases, **68** contained the rare 2*R*,3*R threo* stereochemistry, while **69** possessed an odd-numbered C-29 hydrocarbon chain instead of C-28 found in canonical members of this series. It was a pity to find that rhizochalins C (**68**) and D (**69**) were not active (ED_50_ > 150 μM) against the fluconazole-resistant yeast *C. glabrata* [32]. In 2009, further study on this specimen led to a new antileukemic two-headed sphingolipid rhizochalinin C (**70**), which was the aglycone of rhizochalin C (**68**). In the bioassay, **70** exhibited a cytotoxic effect against THP-1 (IC_50_ = 6.7 µM) but was inactive against JB6 P+ Cl41 and HeLa cell lines [26]. Four years later, Molinski and his co-worker reported the first total synthesis of rhizochalinin C (**70**) [37].

Isorhizochalin (**71**), isolated as its peracetate (**71a**), was a minor bipolar sphingolipid of stereodivergent biogenesis from the *R. incrustata*. Similar to **60**, its absolute stereochemistry was elucidated as 2*S*,3*R*,26*R*,27*R* by analysis of the CD spectrum of its perbenzoate. **71** was a C-2 epimer of **60** with an *erythro* configuration at the glycosylated 2-amino-3-alkanol-*α*-terminus, in contrast to the canonical *threo* configuration for other representatives of this structural group. In the bioassay, its aglycone, isorhizochalinin (**72**), showed cytotoxicity against human leukemia HL-60 and THP-1 cells with IC_50_ values of 2.90 and 2.20 μM, respectively [38].

### 3.2. Ceramides and Cerebrosides

**Figure 10:**
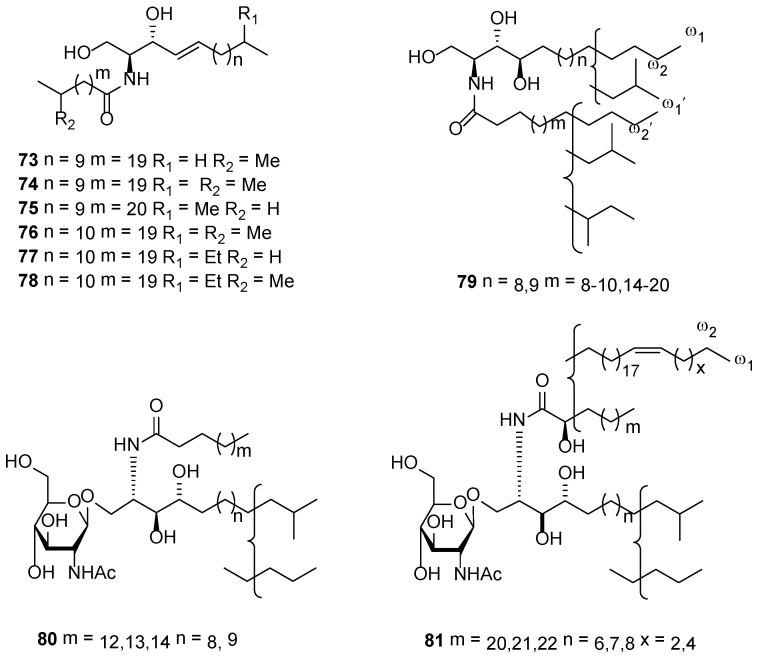
The chemical structures of ceramides and cerebrosides **73**–**81**.

Chemical investigation on the sponge *Oceanapia* cf. *tenuis* collected in Woodin Channel, New Caledonia, led to a series of ceramides, named oceanapins A–F (**73**–**78**) (Figure 10), which were unique for branching at both the sphingosine and fatty-acid chains. A pair of Mosher’s esters of the silyl ether derived from oceanapin D (**76**) was prepared, and the subsequent study on their NMR spectra revealed the 2’*S*,3’*R* absolute configuration for the source compound **76** [39].

An inseparable mixture of ceramides **79** (Figure 10) containing nonbranched and *iso*-branched C_18_ and C_19_-phytosphingosines acylated with nonhydroxylated fatty acids were disclosed in the ethanolic extract of the Australian sponge *Oceanapia* sp. This was the first report of natural *iso*-C_19_-phytosphingosine-containing ceramides. Although no bioactivity was reported for these ceramides, the crude ethanolic extract exhibited antimicrobial activities against *S. aureus*, *B. subtilis*, *E. coli* and *C. albicans* as well as cytotoxic properties against the Erlich murine carcinoma [40]. In another study on the Australian sponge *Oceanapia* sp., two cerebrosides **80** and **81** containing *N*-acetylglucosamine were obtained [41].

### 3.3. Dithiocyanates

**Figure 11:**
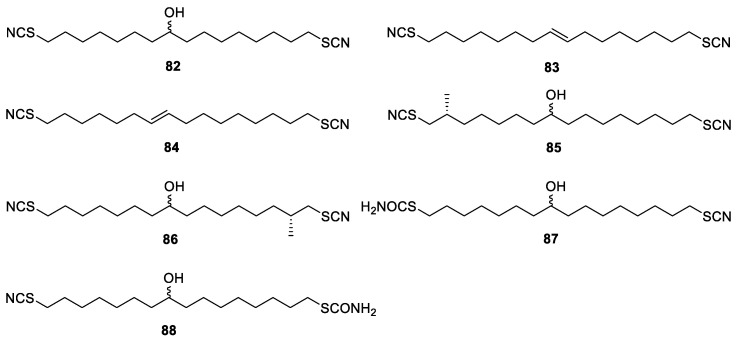
The chemical structures of dithiocyanates **82**–**88**.

The aqueous ethanol extract of an *Oceanapia* sp. collected off the northern Rottnest Shelf, Australia, displayed potent nematocidal activity against the commercial livestock parasite *Haemonchus contortus* (LD_99_ = 135 μg/mL). Bioassay-directed fractionation yielded the bioactive principle component thiocyanatin A (**82**) (LD_99_ = 1.3 μg/mL), together with the inseparable pairs of inactive analogues thiocyanatins B (**83**) and C (**84**), β-methyl branched bisthiocyanates thiocyanatins D1 (**85**) and D2 (**86**) (LD_99_ = 3.1 μg/mL) and thiocarbamate thiocyanates thiocyanatins E1 (**87**) and E2 (**88**) (Figure 11). Their structure assignments were confirmed by chemical synthesis and comparisons with synthetic model compounds. However, the stereochemical character of C-8 in **82** and **85**–**88** remained unknown. In addition to featuring an unprecedented dithiocyanate functionality, thiocyanatins **82**–**88** possessed an unusual 1,16-difunctionalized *n*-hexadecane carbon skeleton and were revealed as a hitherto unknown class of nematocidal agent. Preliminary structure–activity relationship investigations highlighted the importance of both the secondary -OH and -SCN functionalities and the influence of chain length on nematocidal activity [42,43]. 

### 3.4. Polyacetylenes

**Figure 12:**
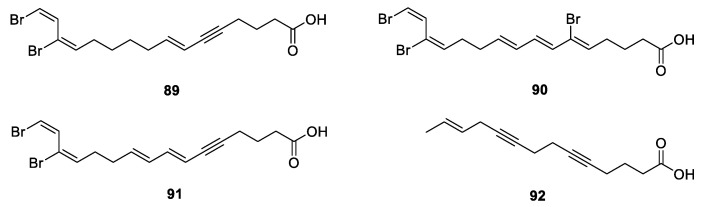
The chemical structures of polyacetylenes **89**–**92**.

The study on the Indonesian sponge *Oceanapia* sp. led to three bromo-substituted polyunsaturated C_16_ fatty acids (7*E*,13*E*,15*Z*)-14,16-dibromohexadeca-7,13,15-trien-5-ynoic acid (**89**), (5*Z*,7*E*,9*E*,13*E*,15*Z*)-6,14,16-tribromohexadeca-5,7,9,13,15-pentaenoic acid (**90**) and (7*E*,9*E*,13*E*,15*Z*)-14,16-dibromohexadeca-7,9,13,15-tetraen-5-ynoic acid (**91**) (Figure 12). Their common structural feature was a (13*E*,15*Z*)-14,16-dibromo-diene terminus. They differed in their C-5 to C-10 segments in unsaturation and halogenation. The cytotoxicity bioassay was tested on the mixture, since compounds **89**–**91** were unstable when pure, which showed only weak cytotoxicity (2+ at 10 μg/mL) against KB cells. Compound **91** showed mild antimicrobial activity against Gram-positive bacteria [44]. A C_14_ acetylenic acid 7*E*,11*E*-tetradecadiene-5,9-diynoic acid (**92**) was isolated as an antimicrobial principle from the sponge of *Oceanapia* sp. collected in Kamagi Bay on the Sada Peninsula. This compound was the first example of a midchain acetylenic acid without a bromine atom, as well as the first reported member of a marine acetylene containing a CH=CH–C≡C–CH=CH–C≡C unit. In the bioassay, **92** exhibited some selectivity in antimicrobial activity. It was moderately active against four mutants of *Saccharomyces cerevisiae* and *C. albicans* but was inactive against *Penicillium chrysogenum* and *Mortierella ramanniana*. It also exhibited inhibitory effects against both Gram-positive and Gram-negative bacteria [45].

## 4. Sterols

**Figure 13:**
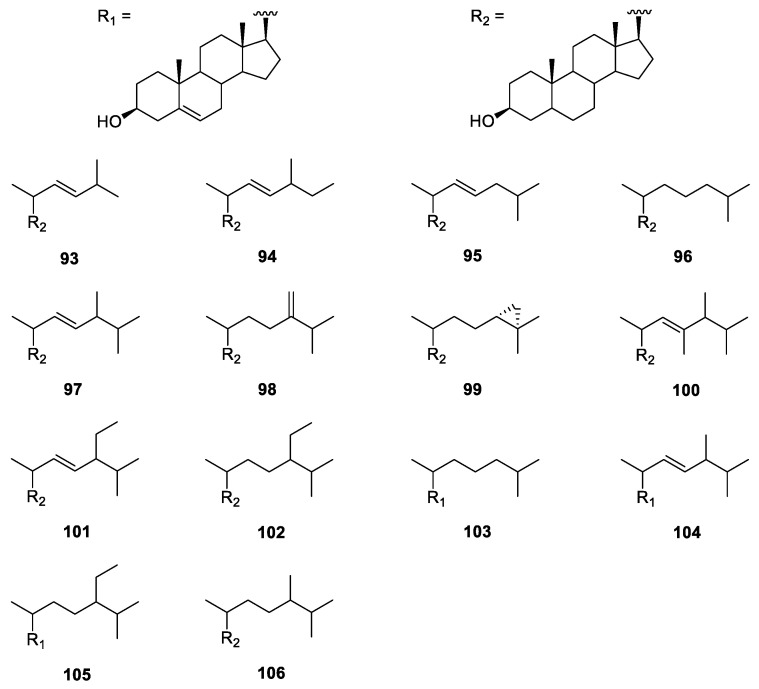
The chemical structures of sterols **93**–**106**.

The sterol profile of a north-western Australian marine sponge *Oceanapia* sp. was reported for the first time by Stonik et al. It contained stanols (**93**–**102**) and △^5^-sterols (**103**–**105**) with 24*R*-24,25-methylene-5*α*-cholestan-3*β*-ol (**99**) (Figure 13) as the main constituent [46]. Notably, the structure of the major cyclopropane-containing stanol **99** was firstly obtained from *Rhizochalina* (= *Oceanapia*) *incrustata* off the coast of the Seychelles Islands [47]. In the investigation on the marine sponge *O*. *sagittaria* from the Gulf of Thailand, 24*α*-methylcholestanol (**106**) was isolated [8].

## 5. Other Miscellaneous

**Figure 14:**
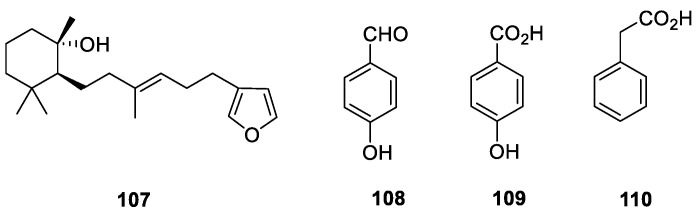
The chemical structures of miscellaneous **107**–**110**.

The organic extract from the Caribbean *O. bartschi* was shown to contain the antibiotic diterpene ambliol A (**107**) (Figure 14) [18]. Three aromatic compounds, *p*-hydroxybenzaldehyde (**108**), *p*-hydroxybenzoic acid (**109**) and phenylacetic acid (**110**), were found in the sponge *O. sagittaria* collected from the Gulf of Thailand [8].

## 6. Chemical Synthesis of Four Secondary Metabolites

### 6.1. Synthesis of (±)-Phloeodictine A1 ((±)-***21***)

**Scheme 1:**
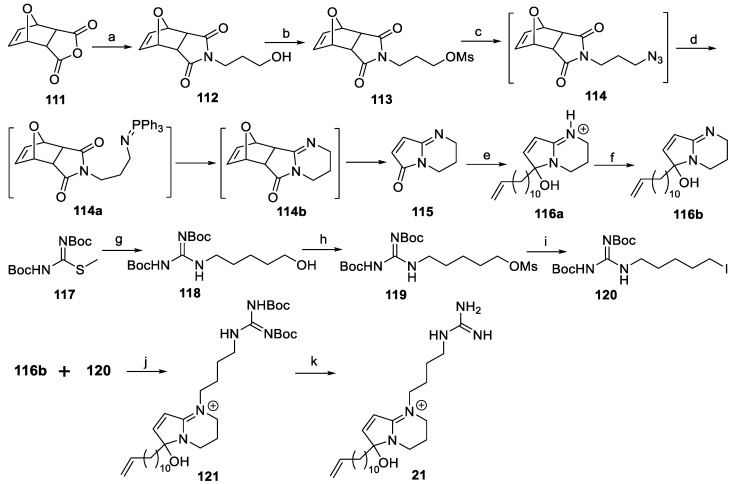
Synthetic route of phloeodictine A1 (**21**). Reagents: (a) 3-aminopropanol, MeOH, 56 °C, 3 d; (b) Et_3_N, MsCl, CH_2_Cl_2_, 0 °C; (c) NaN_3_, DMF, 25 °C, 14 h; (d) Ph_3_P, toluene, reflux 4 h; (e) 11-dodecenyl magnesium bromide, CeCl_3_, THF, 0 °C; (f) 1M NaOH; (g) ω-amino-1-pentanol, THF, 50 °C, 2 h; (h) MsCl, Et_3_N, CH_2_Cl_2_, 0 °C (i) NaI, NaHCO_3_, acetone, reflux, 4 h; (j) DMSO-d_6_, 25 °C, 24 h; (k) TFA/CH_2_Cl_2_ (1:1), 2 h.

The (±)-phloeodictine A1 ((±)-**21**) was synthesized by a convergent route by Snider et al. as shown in Scheme 1 [15]. Furan-maleic anhydride Diels-Alder adduct **111** was used as the starting material for the 6-hydroxy-1,2,3,4-tetrahydropyrrolo[1,2-*a*]pyrimidinium skeleton. Imide **112** was obtained from **111** via a reaction with 3-aminopropanol, which was quantitatively converted to mesylate **113**. The reaction of **113** with NaN_3_ provided azide **114**. The Eguchi aza-Wittig reaction of **114** afforded **114b**, which was followed by a thermal retro Diels–Alder reaction to liberate **115**. The addition of Grignard reagents to **115** produced **116a**. Washing a CH_2_Cl_2_ solution of **116a** with 1 M NaOH solution afforded **116b**. In addition, Snider et al. selected a convergent route for iodide **120** containing a protected guanidine on the other end of the chain. The approach was that the reaction of **117** with the appropriate *ω*-amino-1-alkanol in THF gave **118**, then mesylation and successive displacement with iodide afforded **120**. Finally, alkylation **of 116b** with **120** afforded **121**, and subsequent deprotection of **121** completed the synthesis of **21**.

### 6.2. Synthesis of 6-Br-8-keto-conicamin A *(**55**)*

**Scheme 2:**
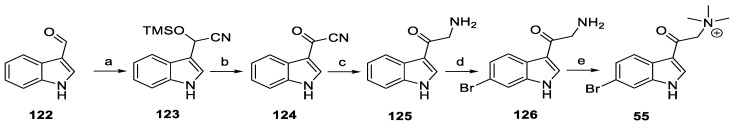
Synthetic route of 6-Br-8-keto-conicamin A (**55**). Reagents: (a): TMSCN, DME, reflux 1.5 h, cool; (b): DDQ (dropwise), Dioxane, rt; (c): H_2_, Pd/C, AcOH; (d): Br_2_, rt 24 h, HCOOH:CH_3_COOH (1:1); (e): Mel, rt 16 h, MeOH, KHCO_3_.

In Crews et al.’s work, indole-3-carboxaldehyde **122** was used as the starting material as outlined in Scheme 2 [19]. First, **122** was esterified to yield its cyanohydrin silylether **123**, then the oxidation of **123** via DDQ followed by hydrogenation led to keto-tryptamine synthon **125**. Bromination of **125** proceeded in a straightforward fashion, producing the bromo-keto-tryptamine **126**. The final step involved the methylation of **126** to afford 6-Br-8-keto-conicamin A (**55**).

### 6.3. Synthesis of Rhizochalinin C *(**70**)*

**Scheme 3:**
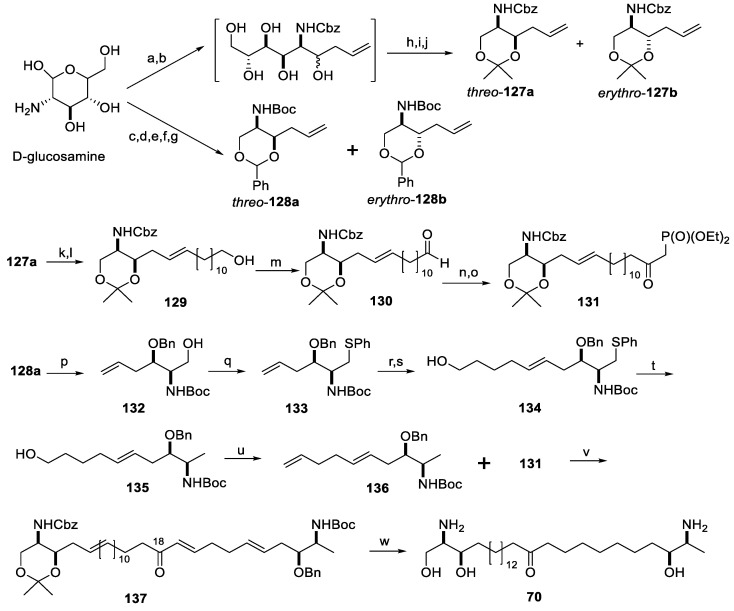
Synthetic route of rhizochalinin C (**70**). Reagents: (a) In^0^, allyl bromide, 1,4-dioxane:H_2_O (3:1), 100 °C; (b) CBz-Cl, NaHCO_3_ (aq.); (c) In^0^, allyl bromide, 1,4-dioxane:H_2_O (3:1), 100 °C; (d) (Boc)_2_O, NaHCO_3_ (aq.); (e) NaIO_4_, H_2_O; (f) NaBH_4_, MeOH; (g)CSA, (MeO)_2_C(CH_3_)_2_; (h) NaIO_4_, H_2_O; (i) NaBH_4_, MeOH; (j) CSA, (MeO)_2_C(CH_3_)_2_; (k) tetradec-13-enyl acetate, Grubbs II catalyst, CH_2_Cl_2_, reflux; (l) NaOMe, MeOH; (m) DMP, CH_2_Cl_2_; (n) (EtO)_2_P(O)CH_3_, n-BuLi, THF, −78 °C; (o) DMP, CH_2_Cl_2_; (p) AlH_3_, Et_2_O, 0 °C; (q) PhSSPh, n-Bu_3_P, THF; (r) 4-penten-1-ylacetate, Grubbs II cat., CH_2_Cl_2_, reflux; (s) NaOMe, MeOH; (t) Ra-Ni; (u) DMP, CH_2_Cl_2_; (v) Ba(OH)_2_, wet THF; (w) 10 M HCl, MeOH, H_2_, 2 atm, Pd−C.

Molinski et al. disclosed an optimized procedure for rapid diastereoselective access to L-*threo*-sphingoid base synthons, using a remarkable one-pot conversion of unprotected D-glucosamine into useful D-serine synthons based on In^0^-mediated allylation. This method was successfully applied to rhizochalinin C (**70**), which was elaborated as shown in Scheme 3 [37].

The allyl-substituted compounds **127a** and **128a** that were procured from the Barbier allylation of D-glucosamine were followed by differential protections of NH_2_ and OH groups. **127a** was subjected to olefin cross-metathesis with tetradec-13-enyl acetate, after methanolysis, to provide the primary alcohol **129**. Then, Dess−Martin oxidation of **129** led to the corresponding aldehyde **130**, which was followed by the addition of the anion derived from diethyl methylphosphonate, and oxidation delivered the *β*-ketophosphonate **131**. The alcohol **132**, the reduction product of **128a**, was transformed into the phenylthio ether to give **133**. Olefin cross-metathesis of **133** with 4-penten-1-yl acetate followed by methanolysis yielded the primary alcohol **134**. Reduction of **134** delivered protected t*hreo*-2-amino-3-alkanol **135**, and the subsequent oxidation of **135** led to the aldehyde **136**. The Horner–Emmons–Wadsworth reaction of aldehyde **131** and **136** under Paterson conditions gave the *α*,*β*-unsaturated ketone **137**, which was deprotected to yield **70**.

### 6.4. Synthesis of Thiocyanatin A *(**82**)*

**Scheme 4:**
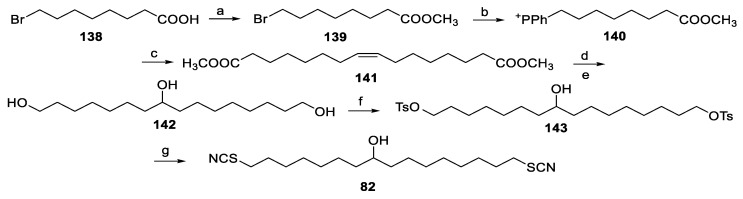
Synthetic route of thiocyanatin A (**82**). Reagents: (a) H_2_SO_4_, MeOH, reflux 16 h; (b) PPh_3_, MeCN, reflux 16 h; (c) NaHMDS, THF/DMPU, O_2_, 60 °C, 16 h; (d) m-CPBA, CH_2_Cl_2_, rt, 16 h; (e) LiAlH_4_, reflux 20 h; (f) p-TsCl, CH_2_Cl_2_, DMAP/NEt_3_, rt, 32 h; (g) KSCN, THF, reflux 16 h.

Capon et al. reported the seven-step total synthesis of thiocyanatin A(**82**) starting from 8-bromooctanoic acid (**138**) [43]. The esterification of 8-bromooctanoic acid (**138**) yielded its methyl ester **139**, which was converted to the Wittig salt **140**. The following one-pot oxidation–Wittig coupling afforded the olefin-diester **141**. The triol **142** was obtained by the epoxidation of **141** with *m*-CPBA and the successive reduction of the corresponding epoxide with LiAlH_4_. Treatment of **142** with TsCl gave the ditosylate **143**. Finally, the displacement of the tosylate groups by thiocyanate afforded racemic thiocyanatin A (**82**) as outlined in Scheme 4.

## 7. Conclusions and Perspectives

A huge library of secondary metabolites was reported from the sponges of the genus *Oceanapia*, with up to 110 compounds with unique structures, from 1989 to July 2019. More than eight species of *Oceanapia* sponges have been chemically investigated, including *Oeanapia sagittaria*, *Oceanapia fistulosa*, *Oceanapia bartschi*, *Phloeodictyon* sp., *Rhizochalina* *incrustata*, *Oceanapia ramsayi*, *Oceanapia phillipensis*, *Oceanupia* cf. *tenuis* and *Oceanapia* sp. The chemical structures were classified as alkaloids, lipids, sterols and other miscellaneous. Among them, alkaloids were encountered most frequently. These compounds exhibited diverse biological properties ranging from insecticidal, cytotoxic and antifeedant to antibacterial. Their unique structures and promising bioactivities have attracted a great deal of attention from synthetic chemists for their total synthesis.
